# Is YouTube a reliable source of health-related information? A systematic review

**DOI:** 10.1186/s12909-022-03446-z

**Published:** 2022-05-19

**Authors:** Wael Osman, Fatma Mohamed, Mohamed Elhassan, Abdulhadi Shoufan

**Affiliations:** 1 Department of Biology, and Pre-Med Program, College of Arts and Sciences, Khalifa University, Abu Dhabi, UAE; 2 Center of Biotechnology, Khalifa University, Abu Dhabi, UAE; 3 Center for Cyber-Physical Systems (C2PS), Khalifa University, Abu Dhabi, UAE; 4 Department of Electrical Engineering and Computer Science, College of Engineering, Khalifa University, Abu Dhabi, UAE; 5 Saint Agnes Medical Center, Fresno, CA USA

**Keywords:** YouTube, Health information, Medical videos, Quality assessment, Bias

## Abstract

**Background:**

YouTube is a valuable source of health-related educational material which can have a profound impact on people’s behaviors and decisions. However, YouTube contains a wide variety of unverified content that may promote unhealthy behaviors and activities. We aim in this systematic review to provide insight into the published literature concerning the quality of health information and educational videos found on YouTube.

**Methods:**

We searched Google Scholar, Medline (through PubMed), EMBASE, Scopus, Direct Science, Web of Science, and ProQuest databases to find all papers on the analysis of medical and health-related content published in English up to August 2020. Based on eligibility criteria, 202 papers were included in our study. We reviewed every article and extracted relevant data such as the number of videos and assessors, the number and type of quality categories, and the recommendations made by the authors. The extracted data from the papers were aggregated using different methods to compile the results.

**Results:**

The total number of videos assessed in the selected articles is 22,300 (median = 94, interquartile range = 50.5–133). The videos were evaluated by one or multiple assessors (median = 2, interquartile range = 1–3). The video quality was assessed by scoring, categorization, or based on creators’ bias. Researchers commonly employed scoring systems that are either standardized (e.g., GQS, DISCERN, and JAMA) or based upon the guidelines and recommendations of professional associations. Results from the aggregation of scoring or categorization data indicate that health-related content on YouTube is of average to below-average quality. The compiled results from bias-based classification show that only 32% of the videos appear neutral toward the health content. Furthermore, the majority of the studies confirmed either negative or no correlation between the quality and popularity of the assessed videos.

**Conclusions:**

YouTube is not a reliable source of medical and health-related information. YouTube’s popularity-driven metrics such as the number of views and likes should not be considered quality indicators. YouTube should improve its ranking and recommender system to promote higher-quality content. One way is to consider expert reviews of medical and health-related videos and to include their assessment data in the ranking algorithm.

**Supplementary Information:**

The online version contains supplementary material available at 10.1186/s12909-022-03446-z.

## Background

YouTube is the world’s second most popular search engine and social media platform [1]. In 2020, YouTube had more than 2.1 billion users, resulting in over one billion hours of video being viewed per day and over 500 hours of video being uploaded each minute [2]. According to published statistics, over 95% of the Internet population are regularly interacting with YouTube in 76 different languages from more than 88 countries [3, 4]. A telephone survey conducted in the United States revealed that more than74% of adults were using YouTube in September 2020 [5].

The growing popularity of YouTube can be attributed to multiple factors. First, users with an internet connection can easily access YouTube’s video service via PCs, laptops, tablets, or mobile phones. More than 70% of YouTube users are accessing online videos through the mobile phone application [6]. This made the YouTube video experience much more enjoyable and available to all users on-demand, anywhere and anytime. Moreover, YouTube is particularly popular among young people who spend hours watching online videos, interacting with others, and sometimes creating their own content [4, 7]. A study conducted in Portugal in 2018 revealed that YouTube is popular among young trainees and residents for surgical preparation [8]. Another aspect that encourages users to utilize YouTube is sharing the enormous advertising revenue with the content creators (also known as YouTubers). This motivates young users to invest in YouTube and spend more time creating and editing online videos.

Nowadays, YouTube has emerged as a valuable educational resource. Specifically, the YouTube model represents a visual model that includes both theoretical and practical knowledge that could be used for teaching purposes. YouTube’s popularity, ease of access, and social nature made it a powerful tool for influencing individuals’ decisions and promoting their well-being.

For example, Mamlin and colleagues (2016) predicted that social media platforms, such as YouTube, would be widely used to (i) exchange healthcare information between healthcare providers and consumers, (ii) facilitate peer-to-peer patient support, and (iii) enhance public health surveillance [9]. The health information videos on YouTube are derived from various sources such as doctors, health institutions, universities and medical schools, patients, and advertisers. However, regardless of the content’s source, YouTube’s terms of service stipulate that “the content is the responsibility of the person or entity that provides it to the Service” [10]. YouTube’s search results are based on popularity, relevancy, and view history rather than content quality. This creates an issue for informal or unguided learners who are increasingly exposed to unverified and partly misleading content that could promote unhealthy habits and activities [11]. For example, a recent study found that more than 25% of the most viewed YouTube videos addressing COVID-19 contained misleading information that reached millions of people worldwide [12]. Furthermore, Nour and colleagues (2016) reported that both accurate and inaccurate YouTube videos discussing psoriasis received similar views [13].

Considering the literature, a limited number of in-depth literature reviews have addressed the content quality issue of healthcare-related videos on YouTube; either in general [14, 15] or in particular to specific topics such as surgical education [16]. Each of these studies has reviewed 7 to 18 articles only. Due to the limited number of reviewed articles, such studies did not provide an extensive analysis of the problem nor a comprehensive discussion of the results and recommendations. Most of these studies highlighted that patient education on YouTube doesn’t follow quality standards and could be misleading.

This paper presents a comprehensive review of the literature related to the content quality of healthcare information of YouTube videos.

## Methods

### Literature search

The search was conducted using Google Scholar, Medline (through PubMed), EMBASE, Scopus, Direct Science, and Web of Science databases from April 1st through April 31st, 2021. ProQuest database was also searched for dissertations and theses to avoid publication bias. As we observed a noticeable shift in the number of publications discussing the COVID-19 pandemic after that, which may, in return, affect our conclusions, we have limited our search to papers published by August 2020 to ensure unbiased coverage of health information topics. We also found that by that time, similar systematic reviews on YouTube COVID-19 pandemics had already been published [12, 17, 18]. We searched several databases for publications that contain the keywords “YouTube” and “quality” in the title and at least one of the following terms “medical, medical education, health, healthcare, health information.” In addition to the common limiters (see eligibility below), we applied the OR and AND Boolean operators to restrict the keyword searches. The searches were conducted independently by two researchers (FA, WO). In case of disagreements, a third researcher (AS) was consulted.

### Eligibility

The inclusion criteria for the papers were: peer-reviewed original articles about the educational quality of YouTube medical videos published between 2005, which is the YouTube’s establishment year, until end of August 2020 in English Language. The exclusion criteria included papers that did not meet the inclusion criteria, duplicate publications, technical reports, organization websites, case reports, and organizational reports.

### Papers selection

In this step, we went through the titles and abstracts of the collected papers to assess them against the inclusion criteria. During this process, we performed an initial annotation of the papers and classified them into three classes: eligible, not eligible, undecided. In some cases, the abstracts were not sufficiently informative, and we couldn’t decide whether to include the papers or not. Thus, we scanned the full texts and the supplementary materials of these papers to decide upon their eligibility.

### Data extraction

In this step, we used a datasheet to record the following information about every publication: title, abstract, topic, quality assessment score and results, number of assessors, number of videos, the resulting categories and classifications, type and source of bias, and conclusion and recommendations. Data extraction and analysis were conducted using the PRISMA recommendations for systematic reviews [19].

### Data synthesis

The reviewed studies followed three schemes for evaluating the content quality on YouTube: scoring-, categorization-, or bias-based evaluation. We performed data synthesis depending on these evaluation schemes. For this purpose, we used simple descriptive statistics such as means, standard deviations, ranges, interquartile ranges, and frequency distribution using histograms. In the case of categorization-based evaluation, the researchers used different numbers and labels for the quality categories. To compile the data from these papers, we created new quality classes and used a heuristic approach to map the categories in the reviewed studies to our classes. Furthermore, we applied qualitative analysis of authors’ recommendations to compile a concise set of general recommendations for improving the quality of medical content on YouTube.

Due to the nature of the studies, it was not possible to discuss the sources of heterogeneity in the data or perform sensitivity analyses, aside from the differences between the languages of the videos analyzed across papers. A challenge we encountered in this study was the inability to apply the normal sources of bias found in clinical trials and research, aside from language bias. There has been no meta-analysis performed.

## Results

### Methodological aspects and general findings

**Fig. 1:**
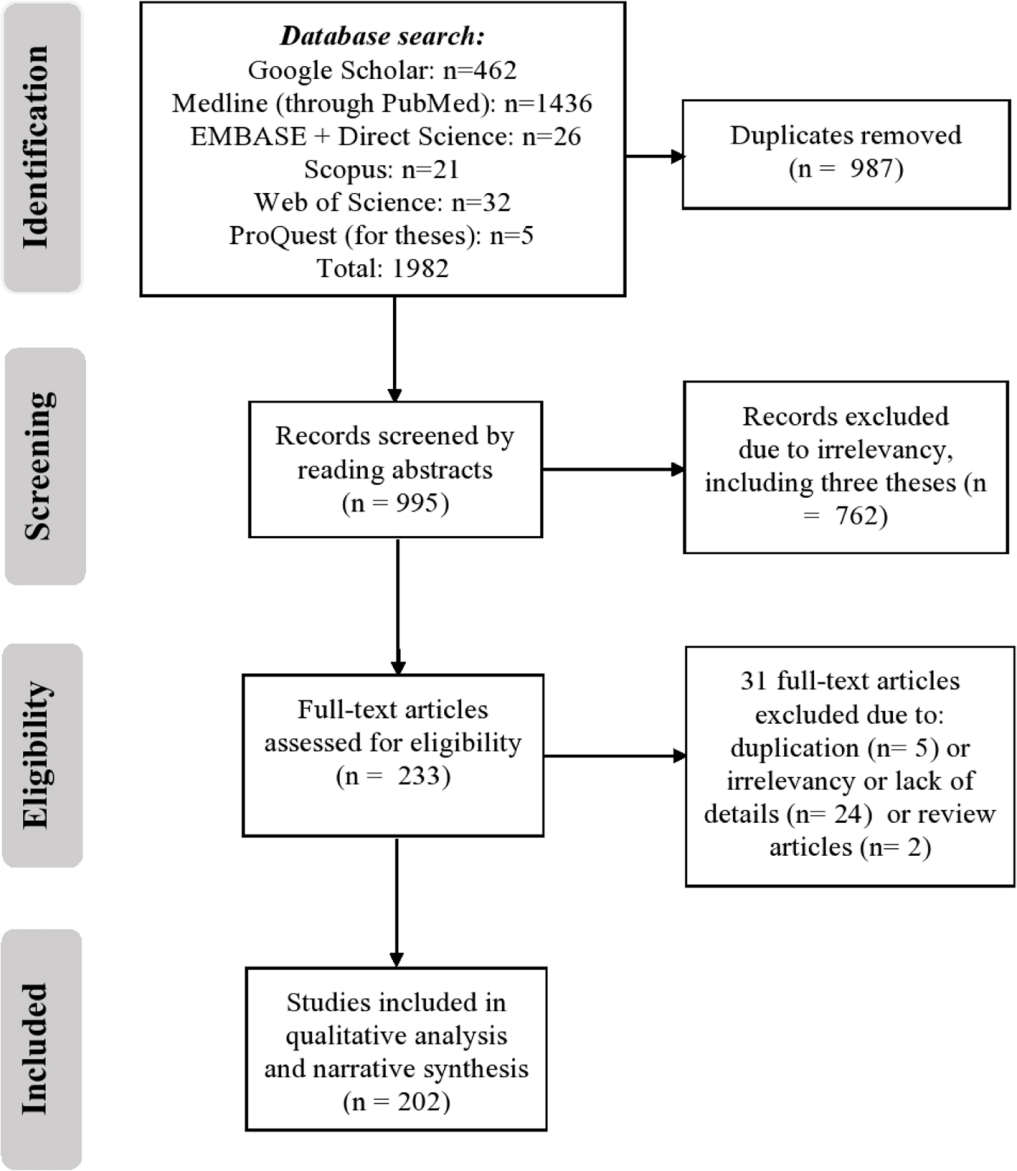
PRISMA Flow chart of literature screening and selection for reviewed articles

Our initial search returned 1982 publications. In this review, the final number of considered studies was 202 articles. In Fig. 1, we present PRISMA flow chart and the approach we followed to come up with the number of 202 eligible papers. In reviewing the full-texts, we reviewed the five theses [20–24]; but we excluded three of them from this study [20–22] since they presented descriptive analyses of health topics without assessing the quality of the research as an eligibility criterion. The titles and topics of all considered articles are summarized in Supplementary Table 1.

We found that the researchers in this field followed the same general approach that is characterized by the following points:

1. Focusing on a single topic, such as a particular disease or treatment.
2. Considering a cross-sectional analysis of the considered videos after identifying the inclusion criteria, such as being in English or having a minimum number of views.
3. Evaluating the considered videos by one or more experts, who are usually among the authors themselves. Researchers in some studies evaluated the scoring reliability using an inter-rater agreement analysis. During this process, discrepancies were resolved by consensus, or by inviting an additional assessor to settle disagreements.
4. Assessors used a scoring system to assess the quality of health-related content. The scoring systems are either self-devised or standardized such as GQS, DISCERN, and JAMA. JAMA is a 4-point scoring system while the other two standards have a 5-point scale.
5. According to the scores, videos were assigned to different quality classes.
6. Depending on the outcome of the scoring or the classification processes, authors gave a general evaluation of the ability of YouTube videos to provide reliable health-related information.
7. Based on this judgment, some general remarks and recommendations were made.

In the following section, we summarize the results of our review considering this general approach. We first highlight some methodological aspects and then compile the findings of reviewed papers.

Table 1, summarizes some statistics related to the number of videos, assessors, and the number of quality categories included in the reviewed studies. The total number of videos in all the reviewed studies that we considered was approximately 22,300 videos. The median number of videos per article was 94 (interquartile range = 50.5–133), and a median of two assessors were involved in each assessment in each article (interquartile range = 1–3). The variation in the number of quality categories poses some difficulty for aggregating the data, as will be discussed later. As an example, some studies classify the videos into three classes, for example “excellent”, “moderate”, and “poor” [25], whereas other studies use four categories, for example “very useful”, “useful”, “slightly useful”, or “misleading” [26].

**Table 1:** The basic metrics for quality assessment of health-related content in studies

Feature	Mean	SD	Median	Q1	Q3	Max	Min
*Number of videos*	115	116	94	50.5	133	1000	2
*Number of assessors*	2.6	1.2	2	1	3	8	1
*Number of quality classes*	3.2	0.9	3			6	2

*Abbreviations*: *SD* Standard deviation, *Q1* First quartile, *Q3* 3rd quartiles

**Fig. 2:**
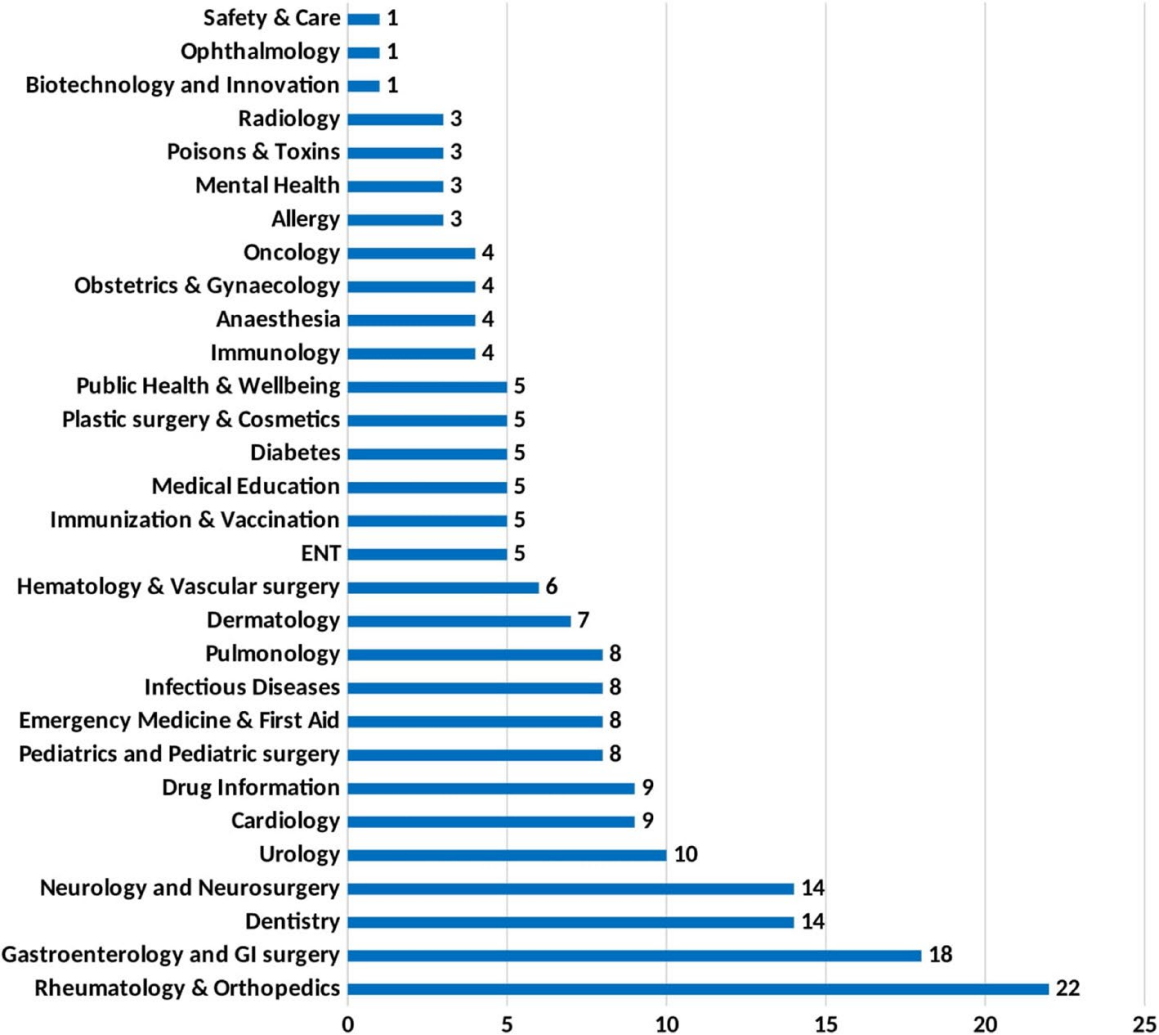
Fields addressed by reviewed studies and number of papers in the respective field

As mentioned before, each reviewed paper examined a series of videos related to a single topic. In a few cases, the same topic was discussed in more than one study. This results in a wide variety of topics included in the reviewed papers. To provide a concise overview, of grouped these topics into 30 different medical categories and determined the number of studies within each category as shown in Fig. 2.

**Fig. 3:**
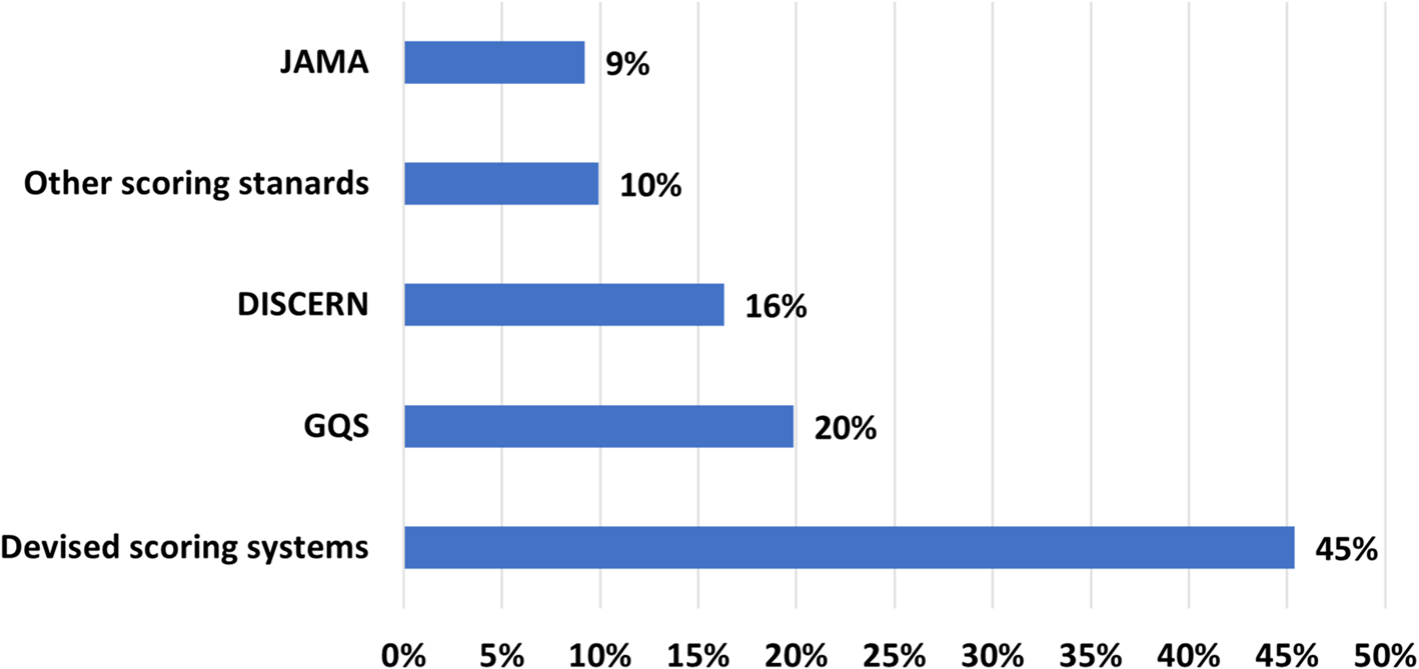
Common scoring systems for assessing the content’s quality

For assessing the content quality, most authors used their scoring systems which are frequently based on some reference in the respective medical field (Fig. 3). For instance, Brooks and colleagues (2014) based their evaluation of videos about patient information for lumbar discectomy on the recommendations of the British Association of Spine Surgeons [27]. Aside from this, many authors adhere to general quality standards. Among these are the Global Quality Standard (GQS), the DISCERN instrument, and the Journal of American Medical Association (JAMA) benchmark criteria. These are described in more detail elsewhere [28, 29].

### Quality assessment results

In the reviewed studies, content quality was assessed through scoring, categorizing, or both. Furthermore, many authors have correlated quality metrics with video popularity metrics such as the number of views and likes. Almost all reviewed papers provided some recommendation statements at the end. The following section compiles the findings of the reviewed studies according to these aspects.

### Quality assessment by scores

**Fig. 4:**
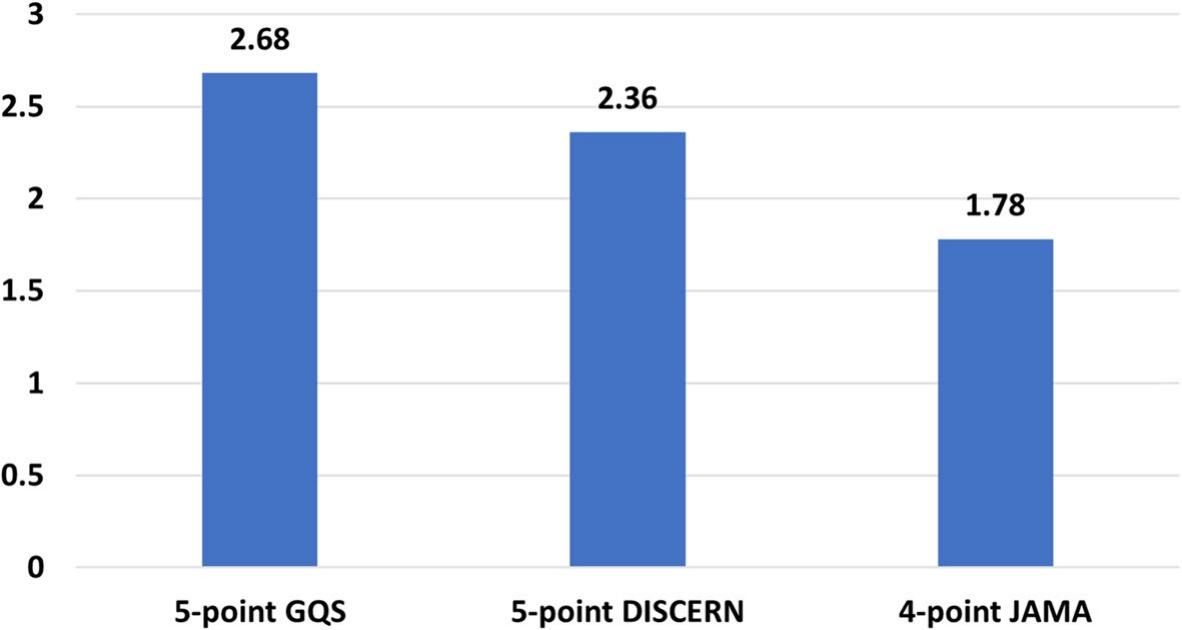
The average quality measures from three general standard scores: GQS, JAMA, and DISCERN

Figure 4 presents the mean quality score of videos according to the three most used standards, GQS, DISCERN, and JAMA. As an example, 25 papers used the GQS standard and provided a mean score for all assessed videos. The value of 2.68 in Fig. 4 is the average of the 25 mean values presented in related papers. As shown in the figure, the mean score is average for all three standards. Note that JAMA is a 4-point scoring system while the other two standards have a 5-point scale.

### Quality assessment by classification

Researchers who evaluated content quality through classification used category labels related to quality, usefulness, accuracy, and reliability. To aggregate the data of these studies, we mapped the used category labels to one of the five labels provided in Table 2. For example, the labels “Excellent”, “Very useful”, “Very accurate,” and “High quality” in the analyzed papers were mapped to the label “Excellent quality” in this paper. In determining the mapping, we relied on a heuristic methodology that took into account the number and intensity of labels in the papers.

**Table 2:** Assigning quality categories in reviewed papers to five categories for the purposes of data aggregation

Excellent quality	Good quality	Average quality	Not useful	Poor quality
- Excellent - Very useful - Very accurate - High quality	- Educationally useful - Useful - Good - Accurate - Reliable	- Fair - Satisfactory - Slightly useful - Somewhat useful - Moderately useful - Intermediate quality	- Not educationally useful - Irrelevant - Offering little value	- Dangerously misleading - Misleading - Very poor - Poor - Inadequate - Low quality - Not reliable - Inaccurate

**Fig. 5:**
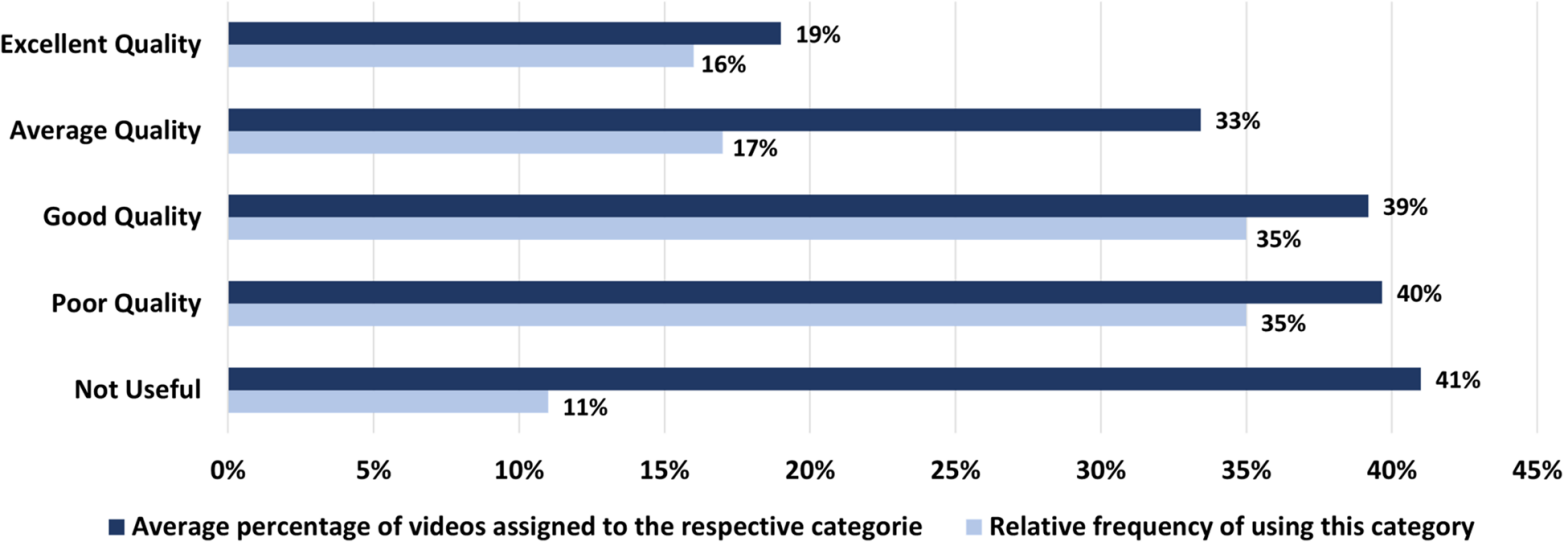
The most common quality categories for the content as used in the reviewed papers

Figure 5 illustrates the results of data aggregation. The upper dark bars represent the average percentage of videos classified into each category. For example, the percentage of 40% in the figure is the average of the percentages of videos, which were assigned to the category “poor” in the reviewed papers. The light bars indicate the relative frequency of using these categories in the related studies. Accordingly, “not useful “ was the most frequently used category, followed closely by “poor quality” and “good quality,” while “excellent quality” was the least frequently used category.

**Fig. 6:**
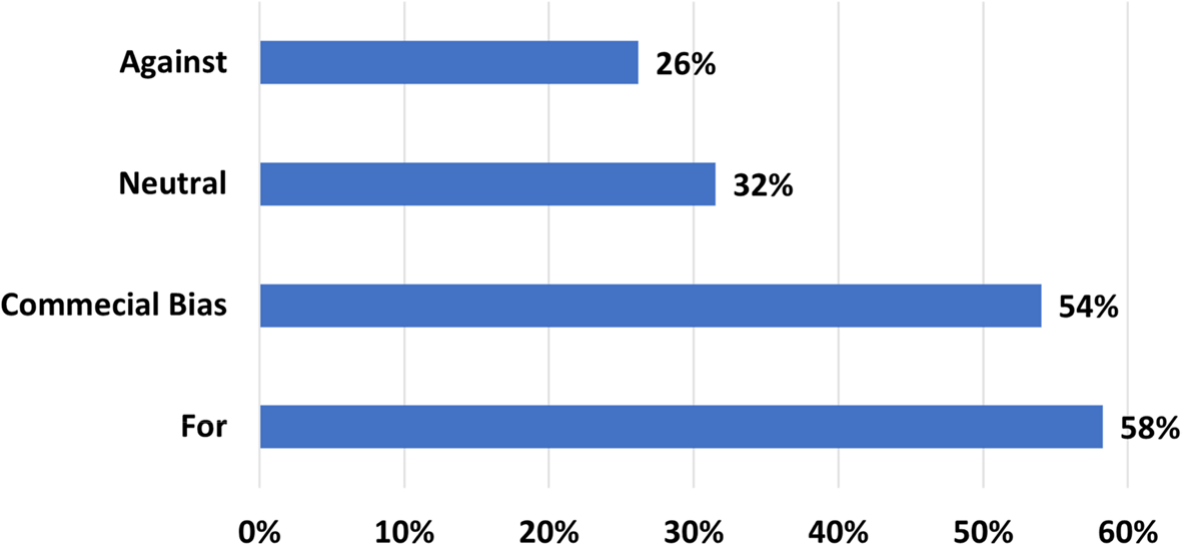
The classification of the reviewed papers according to bias classes

In some papers, controversial topics were discussed, such as vaccination [30] or unauthorized treatments [31]. In such cases, the authors try to classify the videos according to the bias of the producer towards or against the addressed topic. The results are shown in Fig. 6. The figure indicates that 58% of the videos are in support of the treatment discussed. Most of the videos reflect commercial interests (51%), while only 32% are neutral, highlighting the advantages and disadvantages of the presented topics without supporting or devaluing them.

**Fig. 7:**
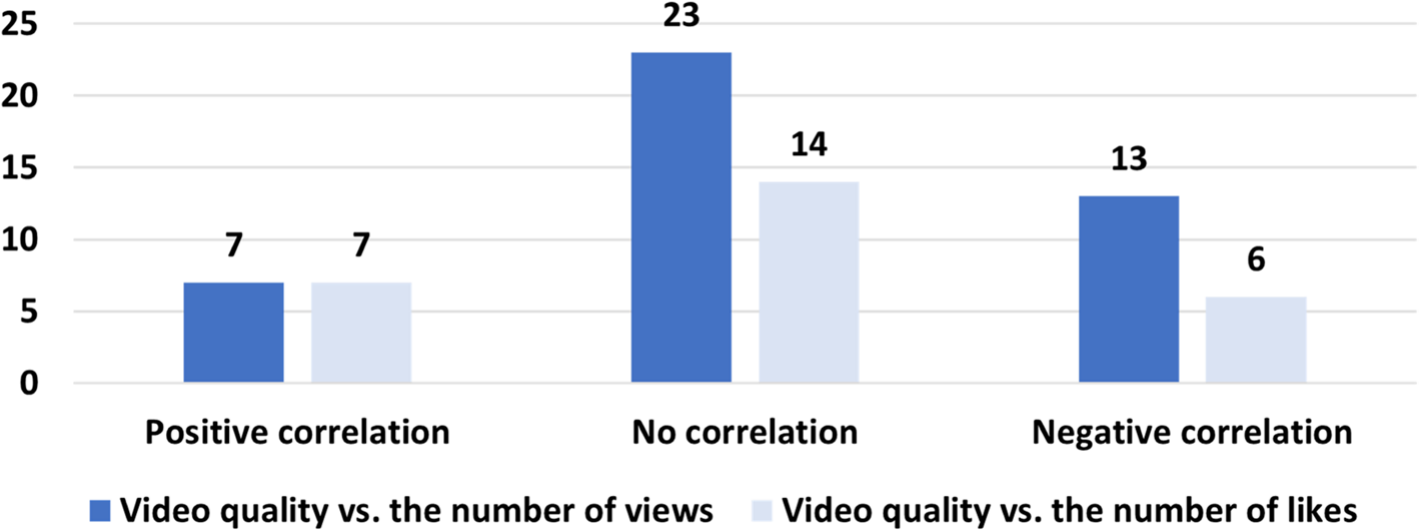
The correlation between video quality and popularity as described in the reviewed papers

Almost one-third of the papers try to correlate the quality of analyzed videos to their popularity metrics, including the number views, likes, dislikes, shares, and comments. Figure 7 summarizes such analyses. For example, the figure shows that 23 papers found no correlation between the number of views and the quality of the videos and 13 found a negative correlation [32, 33]. Negative correlation means that the videos of lower quality were viewed more often than higher-quality videos [34, 35]. Only seven papers found a positive correlation between the quality and popularity in terms of both the number of views and the number of likes [36, 37].

In addition, some papers classified videos according to their comprehensiveness, that is, whether the videos covered all the information that was considered significant for each topic [38–40]. As an example, Pant and colleagues (2012) assessed the credibility of YouTube content on acute myocardial infarction and discovered that only 6% of the reviewed videos addressed all relevant aspects according to the authors’ criteria [41]. The average percentage of comprehensive videos in all reviewed papers is 13.2%.

### Reviewed papers’ recommendations

**Fig. 8:**
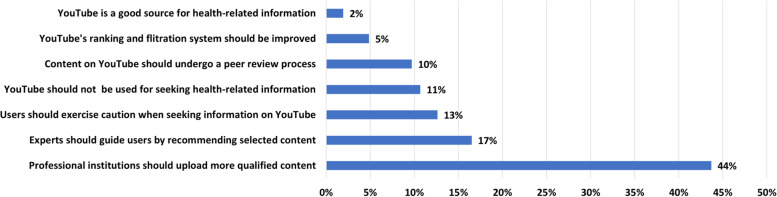
Recommendations derived from the reviewed papers and their frequency

Finally, almost all reviewed papers provide one or more recommendations based on the research findings. Most of these recommendations aim at improving the quality of health-related content on YouTube, as depicted in Fig. 8. Accordingly, 44% of the papers highlight the role of reputable sources such as professional societies, health organizations, academic institutions, medical institutions in providing qualified content on YouTube [42–45].

13% of the papers urge users to be cautious while using YouTube [46, 47] and 17% emphasize that experts should guide users by referring them to high-quality content on YouTube [48, 49]. 10% of the papers suggest that the content on YouTube should be reviewed by experts [50, 51] and 5% recommend that YouTube’s ranking and filtration systems should be improved so that reliable content is presented first, and misleading videos are dropped out [42, 43]. 11% of the reviewed papers explicitly or implicitly regard the situation as irredeemable and entirely discourage using YouTube as a source for health-related information [52–56]. Only a few authors take the opposite position and recommend YouTube without concerns [57, 58].

## Discussion

Good health is a great asset. People seem to place more value on health than on income, career, or education [59]. Human health, however, is vulnerable to diverse internal and external threats inclusive of own behaviors. To avoid, prevent, or mitigate risks to our health, we are dependent on information. Like our health, the quality of this information should be non-negotiable. Unfortunately, however, the ubiquity of digital media is increasingly challenging this principle. This study has shown that YouTube, the most visited media website worldwide, does not only host a significant portion of poor and misleading content but it also promotes these videos through its popularity-based system. So, these videos are accessed and watched by users and patients at least as frequently as good-quality videos [60, 61].

### Why do we have this situation?

YouTube is a business that lives from its viewership and the frequency and duration of access to the platform. To attract more viewers and motivate them to stay longer on the website, YouTube offers growing content and allows any registered member to post videos with almost no restrictions on content quality [17]. This ease of use along with YouTubers’ competition for subscribers, views, likes, and shares has indeed shaped YouTube and contributed to the proliferation of lower-quality content among the search and recommendation lists [62]. On the other hand, the general YouTube user is strongly driven by the entertainment nature of this website and is less skeptical of content quality [63]. This relaxes the demand for verified content from producers. Many consulting agencies are out there to help YouTubers improve the visibility and popularity of their channels [62, 64]. We are not aware of any consultancy specialized in content verification because this remains the sole responsibility of the YouTuber who is seen as the field expert.

### What does this situation mean for the YouTube user?

This systematic review has shown that most researchers are concerned about the quality of medical and health-related information on YouTube. These concerns are serious because watching poor-quality videos can mislead viewers into wrong decisions, practices, or behaviors. However, this cause-effect relationship is under-researched in the literature. So, we don’t yet know whether and how far viewing health-related videos impacts users’ habits, behaviors, decisions. Qualitative studies using surveys and interviews can be helpful in this direction. Indeed, the specific medical field and the urgency of the user’s quest for information can moderate this effect. For example, patients who seek YouTube to learn about the pros and cons of a specific neurological operation towards making a decision are indeed more sensitive to information quality than users who are looking for an effective 5-minute fitness exercise.

### Can the situation of low-quality content on YouTube be resolved or improved?

The analysis of authors’ responses to the current situation showed that many of them urge users to refrain from using YouTube as a source for medical and health-related information. While this avoidance guideline provides the best protection against misleading content, it presents a complete deprivation of the benefits of valuable material on YouTube and disregards the efforts of professional creators who care about content quality. Most of the authors recognize the potential of YouTube and suggest different strategies to overcome the quality challenge. The primary recommendation made by the authors is to encourage recognized professional institutions such as health organizations to be more active in the creation and upload of high-quality content on YouTube. Collaborative efforts to increase the portion of verified content on YouTube are indeed necessary, but is there a guarantee or at least a good chance that such content will appear at the top of search and recommended lists on YouTube? It has been observed that the top-10 video clips in the list returned by the YouTube search engine receive a higher number of views, likes, and comments [65]. The authors attributed this to the preferential attachment process (Yule process), which describes how individuals, who are already wealthy, receive more than those who have less. This indicates that adding new high-quality videos should be accompanied by strategies to promote these videos to appear on the top and become more visible to the user. Such strategies are also in line with the recommendations of some other authors who suggested improving YouTube’s ranking and filtration system. Other strategies to counter the quality challenge address the users who should exercise caution while seeking YouTube for health-related information and consult with experts about this. An efficient way to guide users and patients could be to identify and recommend high-quality channels rather than individual videos [66]. Verified channels can be a reliable point of reference that helps users and patients on a wide range of topics. At the same time, experts should consider the video production styles and strategies that might increase the number of likes per view to promote high-quality content [67]. Finally, many authors recommend that YouTube content should undergo peer review. Expert evaluation is certainly determinant for judging the quality of medical content, but how should evaluation data be made available to YouTube’s viewership? Some efforts have been made to evaluate selected online videos and make them available to students through repositories [68–70]. Csurgeries for example is an excellent educational source which provides a limited set of peer-reviewed videos with surgery content for medical studies [70]. This repository-based approach, however, takes the learner away from YouTube. So, it should be seen as an alternative to YouTube, rather than as a solution for its quality issue. It would be desired if YouTube or intermediate services can provide special interfaces that allow registered experts to review and endorse medical content using general quality standards and employ evaluation data to improve YouTube’s filtration and ranking system [71].

### Important take-home messages for clinical practice

Doctors and patients should exercise caution when using YouTube to access medical information. In addition, physicians should warn patients against relying too much on YouTube. Doctors should also identify and keep a record of high-quality videos and channels related to their fields of study and recommend them to their patients.

### Important take-home messages for clinical research

There is a need for research to identify common features that can be used as quality indicators in health-related videos. Using this information will help users make the appropriate selection. There is also need for research to identify common characteristics that can serve as indicators of health-related video quality. Identifying these characteristics will be helpful in selecting health-related videos.

## Limitations

This study had several potential limitations. First, while mapping the quality categories, specified in the reviewed studies, into quality labels, some minor variations might have occurred. Second, we did not perform sub-analyses for each of the disease classes. Thus, we could not decide whether YouTube’s content about a particular disease had better quality than other content. Despite limiting our search to August 2020 to avoid bias towards COVID-19 content, it may have resulted in a selection bias. We believe that a separate study needs to be conducted on COVID-19 disease and vaccines. It wasn’t possible to apply the sources of bias that are commonly encountered in clinical trials and medical research in our study. The reason is that the research methodology and analyses followed in this study differ from how medical research is usually carried out. However, one possible source of bias in this study is restricting our search to English articles. Despite this, we found that the results of four papers that were not written in English were in line with our results [8, 72–74]. Furthermore, the protocol of this review was not pre-registered for this purpose (e.g., in PROSPERO), which may introduce potential bias per Cochrane guidelines. Initially, we did not register our review in PROSPERO since we noted that similar studies on the same topic were not also registered. However, upon attempting to do that, we were unable to pre-register the application because PROSPERO was overburdened with COVID-19 reviews. Finally, although we included 202 articles in our review, we may have missed some articles that we do not believe will have a significant impact on the study’s findings.

## Conclusions and future work

YouTube is not a reliable source of medical and health-related information. YouTube’s popularity-driven metrics such as the number of views and likes should not be considered quality indicators. YouTube should improve its ranking and recommender system to promote higher-quality content. One way is to consider expert reviews of medical and health-related videos and to include their assessment data in the ranking algorithm.

## Supplementary Information

**Additional file 1.**

## Data Availability

All data is included in the manuscript. Any request about the study design, search strategy, or any other inquiries will be addressed upon contacting the corresponding author.
