# Supplementary material for: Is YouTube a reliable source of health-related information? A systematic review
Source: BMC Med Educ. 2022 May 19;22:382. doi: 10.1186/s12909-022-03446-z (PMC9117585; doi:10.1186/s12909-022-03446-z)
Supplement: Supplementary file 1 — Additional file 1. [file 12909_2022_3446_MOESM1_ESM.pdf]

Supplementary Table 1: The list of papers included in this systematic review

No	Title	Topic	No. of videos	No. of Reviewers
1	Misleading health-related information promoted through video-based social media: anorexia on YouTube	Anorexia	140	3
2	YouTube as a source of information on cardiopulmonary resuscitation	Cardiopulmonary resuscitation (CPR)	52	2
3	YouTube, dentistry, and dental education	Dental education		2
4	How useful is YouTube in learning heart anatomy?	Heart anatomy	294	
5	Vaccine-critical videos on YouTube and their impact on medical students' attitudes about seasonal influenza immunization: a pre and post study	Immunization/vaccination	2	
6	Understanding pharmacokinetics: are YouTube videos a useful learning resource	Pharmacokinetics	48	3
7	YouTube as a potential learning tool to help distinguish tonic-clonic seizures from nonepileptic attacks	Epilepsy, seizures	106	2
8	AB1396 Teaching the millennials: using YouTube for teaching rheumatology in the standard educational settings	Rheumatology - medical education	25	2
9	Comparison of New Era's Education Platforms, YouTube® and WebSurg®, in Sleeve Gastrectomy	Sleeve gastrectomy	10	3
10	YouTube as a Source of Information on Immunization: A Content Analysis	Immunization/vaccination.	153	2
11	YouTube as source of prostate cancer information	Prostate cancer: PSA Testing, radiotherapy, and surgery for	51	2
12	YouTube as a source of information on the H1N1 influenza pandemic	H1N1 influenza	142	2
13	YouTube as a source of information on kidney stone disease	Nephrolithiasis (kidney stones)	199	2
14	YouTube for information on rheumatoid arthritis—a wakeup call?	Rheumatoid arthritis (RA)	102	2
15	YouTube as a source of clinical skills education	Ten common clinical skill related topics	100	3
16	Assessing the credibility of the "YouTube approach" to health information on acute myocardial infarction	Acute myocardial infarction (heart attack)	104	2

17	YouTube videos as a teaching tool and patient resource for infantile spasms	Infantile spasms	100	2
18	YouTube as a source of chronic obstructive pulmonary disease patient education: a social media content analysis	Chronic obstructive pulmonary disease (COPD)	223	2
19	YouTube as a source of quitting smoking information	Smoking cessation	191	3
20	Medical information on YouTube	Immunization/ vaccination		
21	Medical information on the Internet: quality assessment of lumbar puncture and neuroaxial block techniques on YouTube	Lumbar puncture (LP) and spinal anaesthesia (SA)	38	2
22	YouTube: a good source of information on pediatric tonsillectomy?	Pediatric tonsillectomy	156	2
23	YouTube as an information source for pediatric adenotonsillectomy and ear tube surgery	Adenotonsillectomy + ear tube surgery	102	2
24	YouTube as a source of patient information on gallstone disease	Gallstone disease	131	3
25	Learning electrocardiogram on YouTube: how useful is it?	Electrocardiogram (ECG)	119	2
26	YouTube as a source of information on tanning bed use	Tanning (exposing one's skin to the sun in order to achieve a brown or darkened colour)	72	2
27	The quality of video information on burn first aid available on YouTube	Thermal burn first aid	47	2
28	YouTube as a source of information on mouth (oral) cancer	Mouth (oral) cancer	188	
29	YouTube as an information source for femoroacetabular impingement: a systematic review of video content	Femoroacetabular impingement (FAI)	52	2
30	Videos on rhabdomyosarcoma on YouTube: an example of the availability of information on pediatric tumors on the web	Rhabdomyosarcoma and soft-tissue sarcoma	149	3
31	YouTube as a source of information on rhinosinusitis: the good, the bad and the ugly	Rhinosinusitis	100	2
32	YouTube as a patient-information source for root canal treatment	Root canal treatment	60	3

33	Dissemination of misinformative and biased information about prostate cancer on YouTube	Prostate cancer	150	
34	YouTube videos as a source of medical information during the Ebola hemorrhagic fever epidemic	Ebola hemorrhagic fever (EHF)	100	2
35	Is YouTube useful as a source of information for Sjögren's syndrome?	Sjögren's syndrome	70	2
36	Popular on YouTube: A critical appraisal of the educational quality of information regarding asthma.	Asthma	200	5
37	Effectiveness of YouTube as a source of medical information on heart transplantation	Heart transplantation	342	2
38	YouTube as a source of information on Ebola virus disease	Ebola virus disease (EVD	118	2
39	YouTube™ as a source of patient information for lumbar discectomy	Lumbar discectomy	81	2
40	YouTube as a source of useful information on diabetes foot care	Diabetes foot-care		2
41	A qualitative analysis of methotrexate self-injection education videos on YouTube	Self-administer subcutaneous methotrexate	51	3
42	Assessment of YouTube videos as a source of information on medication use in pregnancy	Medication use in pregnancy	314	3
43	YouTube provides irrelevant information for the diagnosis and treatment of hip arthritis	Hip arthritis	133	2
44	Patient information on breast reconstruction in the era of the world wide web. A snapshot analysis of information available on YouTube. Com	Breast cancer	100	
45	YouTube as a source of information on cervical cancer	Cervical cancer	172	2
46	Analysis of YouTube as a source of information for West Nile Virus infection	West Nile Virus infection	106	2
47	Evaluating the accuracy and quality of the information in kyphosis videos shared on YouTube	Kyphosis	50	
48	YouTube as a source of patient information for varicose vein treatment options	Interventional treatment for varicose veins	228	3
49	Is YouTube useful as a source of health information for adults with	Type 2 diabetes	71	5

	type 2 diabetes? A South Asian perspective			
50	A health information recommender system: Enriching YouTube health videos with Medline Plus information by the use of Snomed CT terms	Health education	1000	2
51	Analysis of YouTube as a source of information for peripheral neuropathy	Peripheral neuropathy	200	3
52	Mid-urethral slings on YouTube: quality information on the internet?	Mid-urethral sling (MUS) procedures	56	5
53	YouTube provides poor information regarding anterior cruciate ligament injury and reconstruction	Anterior cruciate ligament (ACL), injury and reconstruction	39	2
54	YouTube as a source of health information: Analysis of sun protection and skin cancer prevention related issues	Tanning	281	4
55	YouTube as a source for parents' education on early childhood caries	Early childhood caries (ECC)	60	2
56	The reliability of YouTube videos in patients education for Glioblastoma Treatment	Glioblastomas	9	3
57	YouTube as a source of information on varicose veins	Varicose veins	281	2
58	Disaster evacuation for persons with special needs: a content analysis of information on YouTube	Disaster preparedness	51	2
59	YouTube videos on botulinum toxin A for wrinkles: a useful resource for patient education	Botulinum toxin type A (BTX-A) for wrinkles	60	2
60	YouTube as a source of quit smoking information for people living with mental illness	Smoking cessation for people with mental illness	40	2
61	YouTube and food allergy: an appraisal of the educational quality of information	Food allergy	300	8
62	Evaluation of the reliability, utility, and quality of the information in sleeve gastrectomy videos shared on open access video sharing platform YouTube	Sleeve gastrectomy procedure	100	3
63	Viewer discretion advised: is YouTube a friend or foe in surgical education?	Laparoscopic cholecystectomy (Gallbladder Removal)	10	3

64	YouTube: are parent-uploaded videos of their unwell children a useful source of medical information for other parents?	Parents health education (e.g. dehydration)	66	4
65	Qualitative analysis of Parkinson's disease information on social media: the case of YouTube™	Parkinson's disease (PD)	100	2
66	English-language videos on YouTube as a source of information on self-administer subcutaneous anti-tumour necrosis factor agent injections	Self-administer subcutaneous anti-tumour necrosis factor (TNF) injections	124	2
67	Exploring the use of entertainment-education YouTube videos focused on infection prevention and control	Hand hygiene	70	1
68	YouTube as a source of information for children with paroxysmal episodes	Paroxysmal episodes (infantile spasms, absence seizures, Sandifer syndrome, sleep myoclonus, and shuddering attacks)	126	5
69	YouTube as a source of information for obstructive sleep apnea	Obstructive sleep apnea (OSA)	48	2
70	YouTube as an information source of spinal anesthesia, epidural anesthesia and combined spinal and epidural anesthesia	Spinal anesthesia, epidural anesthesia, combined spinal epidural anesthesia	40	3
71	YouTube as a source of information about retinitis pigmentosa	Retinitis pigmentosa	162	2
**72**	**Mental health on YouTube: Exploring the potential of interactive media to change knowledge, attitudes and behaviors about mental health**	**Mental health and illness**	**360**	**1 ***
73	Quality of YouTube videos for patient education on how to use asthma inhalers	Using Inhalers for asthma medication	20	
74	YouTube videos for patient education on how to use nasal sprays associated with insufficient reliability	Using nasal spray	26	
75	YouTube as a source of patient information: Assessing quality of information in brachytherapy education videos	Brachytherapy	28	3

76	YouTube videos and the rip current hazard: Swimming in a sea of (mis) information	Rip management (due to drowning)	256	
77	YouTube as a potential source of information on deep venous thrombosis	Deep vein thrombosis	485	2
78	Pain neuroscience education on YouTube	Pain neuroscience	106	2
79	Anterior cervical discectomy and fusion YouTube videos as a source of patient education	Anterior cervical discectomy and fusion (ACDF)	50	2
80	YouTube as a source of information in retinopathy of prematurity	Retinopathy of prematurity (ROP)	100	2
81	Gynecologic cancer Information on YouTube: will women watch advertisements to learn more?	Gynecologic cancer		
82	YouTube videos as a source of information about clinical trials: observational study	Clinical trials	115	
83	A cross-sectional study of YouTube videos as a source of patient information about topical psoriasis therapies	Topical treatment for psoriasis (skin disease)	199	2
84	YouTube videos as a source of palliative care education: A review	Palliative care	84	5
85	YouTube as a source of patient information for trans rectal ultrasound-guided biopsy of the prostate	TRUS biopsies	41	3
86	YouTube as source for vaginal mesh information	Pelvic organ prolapse (POP)	49	
87	Quality assessment of YouTube videos as a source of information on Colonoscopy	Colonoscopy	186	3
88	An evaluation of YouTube in disseminating dementia knowledge to older Chinese in Britain	Dementia	2	
89	Psoriasis and the digital landscape: YouTube as an information source for patients and medical professionals	Treatment of psoriasis	182	1
90	YouTube English videos as a source of information on breast self-examination	Breast self-examination	200	2
91	Cooking with cannabis: the rapid spread of (mis) information on YouTube	Edible marijuana consumption		

92	YouTube as a source of information for patients considering surgery for ulcerative colitis	Surgery for ulcerative colitis (UC)	50	2
93	Who is providing dental education content via YouTube?	Dental education	40	
94	Reliability of YouTube Videos for Patient Education on Food Allergies	Food allergies		
95	Potential Patient Education of YouTube Videos Related to Wisdom Tooth Surgical Removed.	Wisdom tooth surgical removal	92	3
96	YouTube videos as a source of information about immunology for medical students: cross-sectional study	Antigen presentation +Immunoglobulin gene rearrangement	152	2
97	Evaluating YouTube as a source of patient education on the role of the hospitalist: a cross-sectional study	Medical care	102	3
98	Chest tube insertion techniques on YouTube: is social media a reliable source of learning medical skills?	Chest tube insertion techniques	37	3
99	Heart failure videos on YouTube-the good, the bad, and the ugly: A study on the utility and education value for patients, healthcare practitioners and learners	Heart failure	200	
100	Does YouTube provide high-quality resources for patient education on atrial fibrillation ablation?	Catheter ablation in Atrial fibrillation (AF)	111	2
101	Are YouTube videos useful for patient self-education in type 2 diabetes?	Type 2 diabetes self-management	393	2
102	Evaluating YouTube as a source of patient information on Dupuytren's disease	Dupuytren's disease	55	2
103	YouTube as a source of patient information for ankylosing spondylitis exercises	Ankylosing spondylitis	56	2
104	YouTube™ as a Source of Information About Primary Bone Tumours	Primary Bone Tumours	183	1
**105**	**Women's stories of breast cancer: Sharing information through Youtube video blogs**	**Breast cancer**	**79**	**1 ***
106	A critical appraisal of YouTube as a source of information for PET/CT	PET/CT	34	2
107	YouTube is a poor source of patient information for knee arthroplasty and knee osteoarthritis	Knee arthroplasty	106	4

108	‘What is anaphylaxis’: a critical appraisal of the quality of anaphylaxis information on YouTube	Anaphylaxis	292	6
109	English language YouTube videos as a source of lead poisoning-related information: a cross-sectional study	Exposure to lead	100	2
110	Are internet videos useful sources of information during global public health emergencies? A case study of YouTube videos during the 2015--16 Zika virus pandemic	Zika virus	101	2
111	Is YouTube the Future of Patient Information? An Analysis of a Common Urological Surgical Procedure Posted on a Global Video Sharing Website	Urological procedures, Robotic Laparoscopic Prostatectomy	42	2
112	Quality of online video resources concerning patient education for the meniscus: A YouTube-based quality-control study	Menisci (Knee cartilage)	50	
113	YouTube as a source of information on confined space safety	Confined Space safety	220	2
114	Does YouTube provide high quality information? Assessment of secukinumab videos	Secukinumab	53	3
115	YouTube™ as a source of patient information about knee replacement surgery	knee replacement surgery	124	2
116	Quality of information available on YouTube videos pertaining to thyroid cancer	Thyroid cancer	52	2
117	Is YouTube an accurate and reliable source of yellow fever information during outbreaks?	Yellow fever	100	3
118	Analysis of YouTube as a source of information for diabetic foot care	Diabetic foot care	87	2
119	Quality and reliability of YouTube videos as a source of patient information on rhinoplasty	Rhinoplasty	100	2
120	The usefulness of YouTube™ videos as a source of information on burning mouth syndrome	Burning mouth syndrome	114	2
121	Patterns of robotic prostatectomy advertising on the Internet: Quality of the information available to US consumers through the use of Google, Yahoo, Bing, and YouTube.	Robotic prostatectomy for prostate cancer treatment	100	

122	Information content of YouTube videos on orthognathic surgery--Helpful?	Orthognathic surgery (jaw surgery)	55	
123	YouTube™ as a source of information on food poisoning	Food poisoning	160	2
124	Does YouTube™ offer high quality information? Evaluation of accelerated orthodontics videos	Accelerated orthodontic treatment (straightening or moving teeth)	80	1
125	YouTube™ as a source of information for patients undergoing laryngectomy: a thematic analysis	Laryngeal cancer	96	2
126	A systematic evaluation of YouTube as an information source for male infertility	Male infertility	42	
127	Quality and reliability of information available on YouTube videos pertaining to transforaminal lumbar epidural steroid injections	Transforaminal lumbar steroid injection	50	2
128	Moving Beyond the Gym: A Content Analysis of YouTube as an Information Resource for Physical Literacy	Physical literacy	300	2
129	Information on surgical treatment of benign prostatic hyperplasia on YouTube is highly biased and misleading	Lower urinary tract symptoms associated with benign prostatic hyperplasia (LUTS/BPH)	159	3
130	Can You Trust What You Watch?-an Assessment of the Quality of Information about Aesthetic Surgery Available on YouTube	12 most common aesthetic surgical procedures	523	3
131	YouTube for Rapid Sequence Intubation Learning, Is It Reliable?	Rapid sequence intubation (emergency aid)	53	2
132	YouTube resources for synthetic biology education	Synthetic biology		
133	YouTube as a learning tool for four shoulder tests	Physical examinations of the shoulder	200	2
134	YouTube as an education tool for shoulder arthroscopy: Is it the future?	Arthroscopy	20	2
135	Vein Port Access: YouTube as a Tool for Learning Vein Port Access Technique	Vein port access technique	51	1
136	Colonoscopy Videos on YouTube: Are They a Good Source of Patient Education?	Colonoscopy	255	6

137	YouTube as a source of patient education in idiopathic pulmonary fibrosis: a media content analysis	Idiopathic pulmonary fibrosis (IPF)	59	3
138	YouTube™ as a source for patient education about the management of dental avulsion injuries	Emergency management of Traumatic dental injuries)	16	3
139	Evaluation of YouTube as a reliable source for patient education on aortic valve stenosis	Aortic stenosis	85	2
140	Social Media Use in Irritable Bowel Syndrome Patient Education: A Content Analysis of YouTube Videos	Irritable bowel syndrome	30	
141	Assessments of YouTube® information about asthma	Asthma	100	
142	What information does YouTube offer on laparoscopic pyloromyotomy?	Laparoscopic pyloromyotomy	34	2
143	YouTube information about diabetes and oral healthcare	Diabetes mellitus	97	2
144	Misleading information about vaccinations on YouTube	Immunization/vaccination	153	
145	Quality of YouTube patient information on prostate cancer screening	Prostate cancer screening	135	
146	YouTube: A Questionable Source of Information for Information for Patients with Inflammatory Bowel Disease	Inflammatory Bowel Disease	50	2
147	Is the YouTube™ an useful source of information on oral leukoplakia?	Oral leukoplakia	28	2
148	Information Shared on YouTube by Individuals Affected by Long QT Syndrome: A Qualitative Study	Long QT Syndrome (heart rhythm condition)	35	2
149	Pelvic Organ Prolapse on YouTube: Evaluation of Consumer Information	Pelvic organ prolapse	100	5
150	YouTube as a source of patient information for carpal tunnel syndrome	Carpal tunnel syndrome	45	2
151	YouTube® as a Source of Information on Healthy Weight Loss Plans: A Snapshot Analysis of Information Quality	Psoriasis treatment	32	
152	Alzheimer disease health-related information on YouTube: A video reviewing study	Alzheimer disease	537	2
153	YouTube as a source of information about orthodontic clear aligners	Orthodontic clear aligners	100	2

154	YouTube as an information source for intra-articular knee injection	Intra-articular knee injections	55	2
155	YouTube as a Source of Patient Information on Colon Cancer	Colon cancer	84	2
156	Analysis and Assessment of YouTube Uploads as a Source of Information for Oral Sub Mucous Fibrosis.	Oral sub mucous fibrosis	100	2
157	YouTube video analysis as a source of information for patients on impacted canine	Impacted canine and impacted canine surgery	61	3
158	YouTube as a source of information about premature ejaculation treatment	Premature ejaculation	132	2
159	YouTube as an information source for clubfoot: a quality analysis of video content.	Idiopathic clubfoot	42	3
160	YouTube as a source of patient information for abdominal aortic aneurysms	Abdominal aortic aneurysms	51	2
161	Lung cancer screening on YouTube: Difficulty of finding balanced information.	Screening for Lung cancer	124	
162	YouTube as a source of information on Mephedrone the new drug	Mephedrone (drug)	28	2
163	YouTube as a source of information for New Delhi Metallo-β-lactamase-1 superbug gene	Antibiotic resistance	29	2
164	YouTube as a Source of Patient Information for Prenatal Repair of Myelomeningocele	Fetal repair of myelomeningoceles	16	4
165	What information about sudden unexpected death in epilepsy (SUDEP) is available on YouTube?	Sudden unexpected death in epilepsy	113	2
166	An Assessment of YouTube as a Source of Information on Breast Reconstruction Following Mastectomy	Breast reconstruction options following mastectomy	129	2
167	Online Information for Colorectal Cancer Screening: A Content Analysis of YouTube Videos	Colorectal Cancer Screening		2
168	YouTube As A Source Of Patient Information For Knee Arthroplasty And Knee Arthritis	Total knee replacements	106	4
169	YouTube and Eosinophilic Esophagitis: An Assessment of the Educational Quality of Information	Eosinophilic Esophagitis	209	6
170	Quality and Reliability of Information Available on YouTube	Gastroesophageal reflux disease (GERD)	77	2

	and Google Pertaining Gastroesophageal Reflux Disease			
171	Are YouTube videos useful as a source of information for oral care of leukemia patients?	Oral care for leukemia patients	80	
172	YouTube as a Patient-Information Source for Cleft Lip and Palate	Treatment of cleft lip and palate.	50	2
173	Assessment of the Quality and Reliability of the Information on Rotator Cuff Repair on YouTube	Rotator cuff (RC) repair surgery.	67	2
174	YouTube as a source of health information: Analysis of sun protection and skin cancer prevention	Sun protection and skin cancer prevention, tanning	281	4
175	'Should I vaccinate my child? 'comparing the displayed stances of vaccine information retrieved from Google, Facebook and YouTube	Immunization/vaccination.	20	
176	YouTube as a Source of Information About the Posterior Cruciate Ligament: A Content-Quality and Reliability Analysis	Posterior cruciate ligament (PCL) of the knee	50	
177	Can You Trust What You Watch? An Assessment of the Quality of Information About Aesthetic Surgery on YouTube	Aesthetic surgery	523	3
178	Effective Use of Popular Internet Video Broadcast Site YouTube for Dissemination of Information about the Potential Pandemic of H1N1 Influenza	H1N1 influenza (swine flu)	142	2
179	A quality assessment of atrial fibrillation catheter ablation material on YouTube; do they contain sufficient information to act as an appropriate patient resource?	Catheter ablation	111	2
180	Online social media platforms and public health information: an exploration into arthritis related videos on YouTube in 2017	Arthritis	63	2
181	Critique Of YouTube As A Data Source For The Qualitative Exploration Of Patient Reported Information On Acute Myeloid Leukemia.	Acute Myeloid Leukemia (AML)	28	
182	Utility Evaluation of Information from YouTube on Breastfeeding for Preterm Babies	Breastfeeding for preterm babies	78	

183	Educational value of surgical videos on YouTube: quality assessment of laparoscopic appendectomy videos by senior surgeons vs. novice trainees	Laparoscopic appendectomy	25	6
184	YouTube as an informational source for brachial plexus blocks: evaluation of content and educational value	Brachial plexus block	86	3
185	Evaluation of the reliability, utility, and quality of the information in cardiopulmonary resuscitation videos shared on Open access video sharing platform YouTube	Cardiopulmonary resuscitation (CPR)	100	2
186	Assessment of "YouTube" Content for Distal Radius Fracture Immobilization	Distal radius fracture immobilization	16	2
187	Evaluating YouTube as a Resource for Trigeminal Neuralgia Patient Education	Trigeminal neuralgia	80	2
188	YouTube as a Source of Information for Transcranial Magnetic Stimulation in Stroke: A Quality, Reliability and Accuracy Analysis	Transcranial Magnetic Stimulation in Stroke	21	2
189	Assessing the educational quality of 'YouTube' videos for facelifts	Facelifts	13	3
190	YouTube as a Potential Training Resource for Laparoscopic Fundoplication	Laparoscopic fundoplication	71	4
191	An Evaluation of Educational Neurological Eye Movement Disorder Videos Posted on Internet Video Sharing Sites.	Neurological eye movement disorder	354	
192	A critical review of obstetric and gynecological physical examination videos available on YouTube: Content analysis and user engagement evaluation	Obstetric and gynecological physical examination	84	2
193	A quality assessment of cardiac auscultation material on YouTube.	Cardiac auscultation	22	2
194	An appraisal of the current and potential value of Web 2.0 contributions to continuing education in oral implantology.	Oral implantology	97	
195	Quality and Reliability of YouTube for Patient Information on Neurotoxins	Neurotoxins	61	2

196	Reliability and Educational Features of YouTube Videos about Hernia Operations Performed Using Laparoscopic TEP Method	Hernia operations	50	
197	Medical Professionals' Review of YouTube Videos Pertaining to Exercises for the Constipation Relief	Pertaining to Exercises for the Constipation Relief	20	8
198	Educational quality of YouTube videos on knee arthrocentesis	Knee arthrocentesis	13	2
199	Educational value of surgical videos on transabdominal pre-peritoneal hernia repair (TAPP) on YouTube	Transabdominal pre-peritoneal hernia repair (TAPP)	20	5
200	Quality of Erector Spinae Plane Block Educational Videos on a Popular video-sharing Platform	Erector spinae plane block	21	3
201	Popular on YouTube: A critical appraisal of the educational quality of information regarding asthma	Asthma	200	5
202	Consulting "Dr. YouTube": an objective evaluation of hypospadias videos on a popular video-sharing website	Hypospadias	53	2

*Articles in bold (# 72 and 105) are theses
